# High-Sensitivity Top-Down Proteomics Reveals Enhanced Maturation of Micropatterned Induced Pluripotent Stem Cell-Derived Cardiomyocytes

**DOI:** 10.1021/acs.jproteome.5c00505

**Published:** 2025-08-26

**Authors:** Mallory C. Wilson, Mitchell Josvai, Janay K. Walters, Jodi Lawson, Kalina J. Rossler, Zhan Gao, Yanlong Zhu, Timothy J. Kamp, Wendy C. Crone, Lee L. Eckhardt, Ying Ge

**Affiliations:** † Department of Chemistry, 5228 University of Wisconsin-Madison, Madison, Wisconsin 53706, United States; ‡ Department of Cell and Regenerative Biology, 5228 University of Wisconsin-Madison, Madison, Wisconsin 53705, United States; § Department of Biomedical Engineering, 5228 University of Wisconsin-Madison, Madison, Wisconsin 53706, United States; ∥ Department of Medicine, School of Medicine and Public Health, 5228 University of Wisconsin-Madison, Madison, Wisconsin 53705, United States; ⊥ Molecular and Cellular Training Program, School of Medicine and Public Health, 5228 University of Wisconsin-Madison, Madison, Wisconsin 53705, United States; # Human Proteomics Program, School of Medicine and Public Health, 5228 University of Wisconsin-Madison, Madison, Wisconsin 53705, United States; ∇ Department of Nuclear Engineering and Engineering Physics, 5228 University of Wisconsin-Madison, Madison, Wisconsin 53706, United States; ○ Department of Mechanical Engineering, 5228 University of Wisconsin-Madison, Madison, Wisconsin 53706, United States

**Keywords:** Top-down proteomics, hiPSC-CM maturation, sensitivity, cardiac model, arrhythmia

## Abstract

Human induced pluripotent stem cell-derived cardiomyocytes (hiPSC-CMs) are increasingly used for disease modeling, drug discovery, and precision medicine, yet their utility is often limited by their immature phenotype. One promising maturation strategy involves using micropatterned substrates that mimic native cardiomyocytes’ organizational growth and stiffness. However, the maturity of this model has not fully been assessed, and there is currently no method to extract proteins from micropatterned hiPSC-CMs for top-down proteomic analysis. Herein, we present a high-sensitivity protein extraction protocol for top-down proteomic analysis of hiPSC-CMs. Through this method, we assessed the maturation of micropatterned hiPSC-CMs compared to traditional monoculture and coculture monolayers at the proteoform level. We found that micropatterned hiPSC-CMs display molecular signatures of cardiomyocyte maturation including increased expression of ventricular myosin light chain isoforms, reduced expression of the fetal troponin T isoform, and decreased phosphorylation of alpha-tropomyosin. This high-sensitivity approach enables robust top-down proteomics from limited, heterogeneous cell populations and identifies the micropattern hiPSC-CM as a more adult-like CM model, broadening the utility of structured culture systems for cardiac disease modeling and translational research. Source data for this manuscript are available via MassIVE repository at massive.ucsd.edu with identifier: MSV000097864.

## Graphical abstract

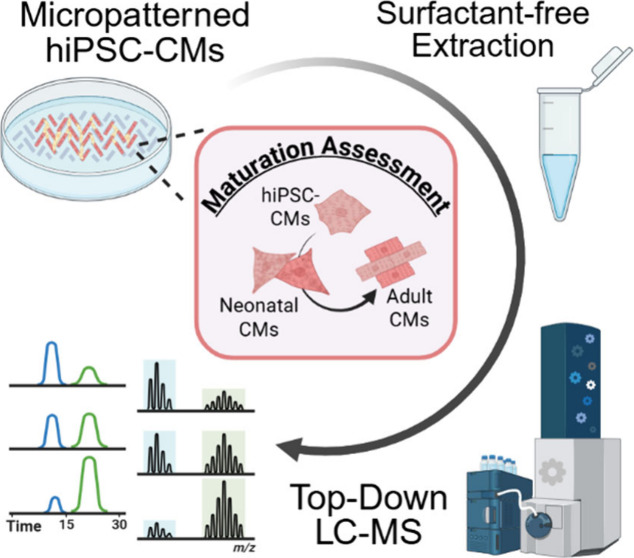

## Introduction

Over the last two decades, human induced pluripotent stem cell-derived cardiomyocytes (hiPSC-CMs) have rapidly grown in popularity for disease modeling, drug discovery, and precision medicine.^1–5^ By enabling a personalized “disease-in-a-dish” approach, these *in vitro* models have revolutionized cardiovascular research toward understanding cardiac diseases and assessing cardiotoxicity directly from patients’ own cells.^6–9^ However, hiPSC-CM models are still limited by their relative immaturity compared to that of adult cardiomyocytes. This is largely due to the difficulty in replicating complex *in vivo* maturation cues and the extended duration required for cardiomyocyte maturation in humans.^10,11^ As a result, hiPSC-CM models often lack the contractile properties, electrophysiology, metabolism, and protein expression necessary to accurately represent an adult patient’s disease phenotype.^3,5,9^

A variety of strategies have been explored to promote hiPSC-CM maturation, including metabolic, electrical, and mechanical conditioning and microengineered platforms.^12–14^ A promising strategy toward maturation is to culture hiPSC-CMs on micropatterned substrates that mimic biological conditions by aligning seeded cardiomyocytes along interconnected lanes to more accurately reflect their *in vivo* geometry than a traditional 2D monolayer.^15,16^ Recently, polydimethylsiloxane (PDMS)-based micropatterns have combined reduced substrate stiffness, chevron lanes, and coculture alongside hiPSC-derived fibroblasts (hiPSC-CFs) to promote native-like development after just 30 days of culture time.^17–19^ However, the molecular maturity of hiPSC-CMs cultured on these micropatterned substrates remains unexplored, leaving a gap in our understanding of the applicability of these models.

Proteoforms—the distinct molecular forms arising from genetic variation, alternative splicing isoforms, and post-translational modifications (PTMs)—play critical roles in defining cellular function and phenotype.^20–22^ Top-down proteomics,^22–26^ which analyzes intact proteoforms, provides an unbiased method to assess cardiomyocyte maturation by capturing changes in protein isoform expression and PTMs.^10,27,28^ Previous studies have identified these markers of maturation by examining sarcomeric protein transitions, particularly the switch from fetal to adult isoforms,^10,29,30^ but detecting these transitions with top-down proteomics has traditionally required a large number of hiPSC-CMs (>1 million cells).^10,27^ Micropatterned hiPSC-CM cultures yield limited cell numbers much lower than previous models (∼50,000 hiPSC-CMs),^16,17^ necessitating the development of sensitive top-down methods to enable the proteoform-level maturation assessment of these models.

Herein, we present a highly sensitive, surfactant-free, top-down proteomics platform that enables robust assessment of hiPSC-CM maturation from limited cell numbers cultured on PDMS micropatterns. With this optimized protocol, we achieve quantitative top-down proteomics of sarcomeric proteoforms directly from micropatterned hiPSC-CMs for the first time. Our findings reveal that micropatterning of a soft substrate markedly accelerates cardiomyocyte proteomic maturation, with isoform switching and changes in the relative abundance of proteoforms comparable to 3D cultures maintained for twice as long. This advancement establishes a robust strategy for hiPSC-CM proteomic analysis from limited cell input, enabling the assessment of functionally mature models with improved reproducibility and translational relevance for disease modeling and drug discovery.

## Experimental Procedures

### hiPSC-CM Culture & Harvest

A full description of the hiPSC to hiPSC-CM and hiPSC-CF differentiation protocols using the small molecule GiWi method^31^ and GIF protocol,^2^ respectively, can be found in the Supplemental Methods. A visual representation of the experimental timeline can be found in the Supporting Information (*Supplementary* Figure S1). After differentiation and magnetic-activated cell sorting (MACS, Miltenyi) purification (Day 16), hiPSC-CMs were split between monoculture monolayers (MM), coculture monolayers (CC) (hiPSC-CMs + hiPSC-CFs), and coculture micropatterns (μP). MM hiPSC-CMs were cultured on PDMS-coated tissue culture plastic (TCP) until Day 30. CC hiPSC-CMs were seeded at a 10:1 ratio of cardiomyocytes to fibroblasts on PDMS-coated TCP and maintained for 14 days (Day 30 total culture time). μP hiPSC-CMs were seeded at a 10:1 ratio of cardiomyocytes to fibroblasts on micropatterned PDMS and cultured until Day 14 (Day 30 total culture time). Cells were harvested and singularized by TrypLE 10X (Life Technologies) incubation for 15 min followed by an immediate trypsin quench via dilution with EB20 medium (Supplemental Methods). To deplete hiPSC-CFs, heterogeneous solubilized cells were incubated on uncoated TCP for 60 min at 37 °C. The supernatant was removed, yielding a pseudoenrichment of hiPSC-CMs and leaving hiPSC-CFs attached to the uncoated TCP. Though hiPSC-CFs were not present in the MM cohort, those samples were also subjected to a 60 min incubation and subsequent supernatant removal to ensure treatment consistency. Enriched hiPSC-CM samples were centrifuged at (100 × g, 10 min, 25 °C), then washed with Dulbecco’s phosphate-buffered saline (DPBS, Thermo Fisher Scientific). Live cell counts were performed using a hemocytometer. DPBS-solubilized monolayer samples were aliquoted to 50k cells/sample to match individual micropattern cell counts. All samples were moved to Protein LoBind centrifuge tubes (Eppendorf), and cells were pelleted via centrifugation (21,000 × g, 15 min, 4 °C). The supernatant was removed, and the samples were stored at −80 °C until protein extraction.

### Surfactant-Free Protein Extraction

The protein extraction protocol was inspired by the hexafluoroisopropanol (HFIP)-enabled single muscle fiber extraction.^32^ On the day of mass spectral analysis, cell pellets were thawed (∼1 min) and resuspended in 20 μL of 90% HFIP extraction solution (90% HFIP, 10 mM l-methionine, 1× HALT protease and phosphatase inhibitor cocktail) via gentle up-and-down pipetting before a 15 min incubation on ice. Samples were then diluted with 20 μL of 0.2% formic acid (FA) and briefly dipped (<2 s) into an ultrasonic water bath three times, followed by three freeze–thaw cycles (incubation at −80 °C for 10 min followed immediately by incubation for 1 min at 37 °C). Gentle agitation was applied between each freeze–thaw cycle. Samples were centrifuged (21,000 × *g*, 15 min, 4 °C), and the supernatant was removed for desalting via buffer exchange. Buffer exchange with 0.2% FA was performed using an Amicon 10 kDa molecular weight cutoff spin filter (MilliporeSigma) spun at 15,000 × *g* (5 × 5 min, 4 °C), then samples were further concentrated to a final volume of around 35 μL (15,000 × *g*, 20 min, 4 °C). Sample concentrations were quantified by using a standard Bradford assay, and the remaining lysate was transferred to an HPLC vial.

### Liquid Chromatography & Mass Spectrometry

OtofControl 3.4 (Bruker Daltonics) was used to collect all LC-MS data. Protein separation was performed using reversed-phase chromatography with a NanoAcuity Ultra-High-Pressure LC system (Waters, Milford, MA, USA). Injection volumes were adjusted to enable 500 ng of total protein. Elution was performed from a home-packed C4 column (200 × 0.250 mm, 2.7 μm, 1000 Å C4 (Halo)) held at 50 °C with a flow rate of 3 μL/min. Mobile phase A (MPA) contained 0.2% FA in H_2_O, and mobile phase B (MPB) contained 0.2% FA in ACN, with a 65 min RPC gradient of the following MPB concentrations: start at 10% MPB, hold 10% until 5 min, 25% at 15 min, 40% at 35 min, 50% at 45 min, 95% at 55 min, adjusted back to 10% at 55.1 min, and held at 10% until 65 min. Eluted proteins were electrosprayed into a high-resolution Impact II quadrupole time-of-flight (QTOF) mass spectrometer (Bruker Daltonics, Bremen, Germany). The end plate offset was set to 500 V, and the capillary voltage was set to 4500 V. The nebulizer was set to 0.5 bar with a dry gas flow rate of 4.0 L/min at 200 °C. Mass spectra were collected at a scan rate of 1 Hz over 300–3000 *m*/*z* with a quadrupole low mass set to 650 *m*/*z*.

### Data Analysis

LC-MS data was processed and analyzed using DataAnalysis (v4.3, Bruker Daltonics) software.^33^ Maximum Entropy Deconvolution of spectra was performed with a resolving power of 60,000 for isotopically resolved proteins. The top 5–7 most abundant charge state ions from the nondeconvoluted spectra were used to produce extracted ion chromatograms (EICs), and relative abundances of isoforms were measured using the ratios of the area under the curve (AUC) of each isoform’s EIC. Relative quantification of proteoforms was performed by deconvoluting mass spectra using the Maximum Entropy Deconvolution algorithm and taking the ratio of the highest peak intensity of the proteoform to the summed intensities of all proteoforms of that protein. Total phosphorylation of proteins with multiple phosphorylation sites was calculated as the ratio of the sum of the peak intensities of all phosphorylated proteoforms (multiplied by the integer number of phosphorylated sites on that proteoform) to the sum of all proteoform peak intensities of that protein. The sophisticated numerical annotation procedure (SNAP) algorithm was applied to determine the monoisotopic masses of all observed ions. Normality was determined using the linearity of Q-Q plots and the Shapiro-Wilke test. Statistical analysis was performed using one-way Analysis of Variance (ANOVA) to compare the means of all three cohorts. Post hoc analysis was performed using Tukey’s Honest Significance Difference (HSD) test for pairwise comparisons; “n.s.” indicates a *p* > 0.05, * indicates *p* < 0.05, and ** indicates a *p* < 0.001.

## Results and Discussion

### High-Sensitivity Top-down Proteomics for Micropatterned hiPSC-CMs Enabled by Surfactant-Free Extraction

First, we aim to establish a high-sensitivity, surfactant-free protein extraction method for samples with limited numbers of hiPSC-CMs inspired by our previously developed high-sensitivity top-down proteomics method for single muscle cells.^32^ The original surfactant-free protein extraction protocol^29^ successfully extracted proteins from skeletal muscle cells using just 25% hexafluoroisopropanol (HFIP), a weakly acidic fluoroalcohol that clusters similarly to surfactants in aqueous solutions.^34^ However, it did not provide efficient extraction of proteins from hiPSC-CM samples, potentially due to differences in the membrane composition, extracellular matrix laid during time in culture, and/or intercellular junctions, like intercalated discs.^35^ To optimize the extraction for the micropattern platform, we increased the concentration of HFIP to 90% and incorporated pulse sonication by brief sample tube immersion in a sonicating water bath (Supplementary Figure S2). Though these strategies alone were not effective solutions, their combination achieved robust cell lysis and solubilization of proteins, facilitating the highly reproducible proteoform-level quantitative analysis of sarcomeric proteins from micropatterned hiPSC-CMs. This is demonstrated by consistent retention times and spectral intensities of three LC-MS technical replicates and consistent linear response of varied protein loading injection replicates (Supplementary Figure S3).

Next, we applied the optimized surfactant-free extraction protocol to hiPSC-CM samples cultured under three distinct conditions (n = 8 per group): monoculture monolayers (MMs), coculture monolayers with hiPSC-CFs (CCs), and cocultures on micropatterned PDMS (μPs) (Figure 1, Figure 2A). Prior to protein extraction, hiPSC-CFs were selectively depleted from samples using a method adapted from protocols for the selective adhesion of primary fibroblasts from tissue (Supplemental Methods).^36^ Briefly, solubilized samples were incubated on uncoated TCP wells for 1 h. Due to their limited adherence capacity,^36^ the cardiomyocytes did not attach to the uncoated TCP in such short time periods and remained in suspension while the fibroblasts adhered to the TCP surface. This process yielded enriched hiPSC-CM samples of approximately 50,000 cells to be treated with the optimized protocol. On the day of LC-MS analysis, proteins were extracted from the cell pellets by the addition of 20 μL of the 90% HFIP extraction solution, generating complex, whole-cell lysates with 269 detected proteoforms (Table S1), including identified sarcomere proteoforms (Table S2). Notably, with just 50,000 cells, we are able to achieve comparable coverage of intact sarcomere proteins as in previous studies.^10,28^

**Figure 1:**
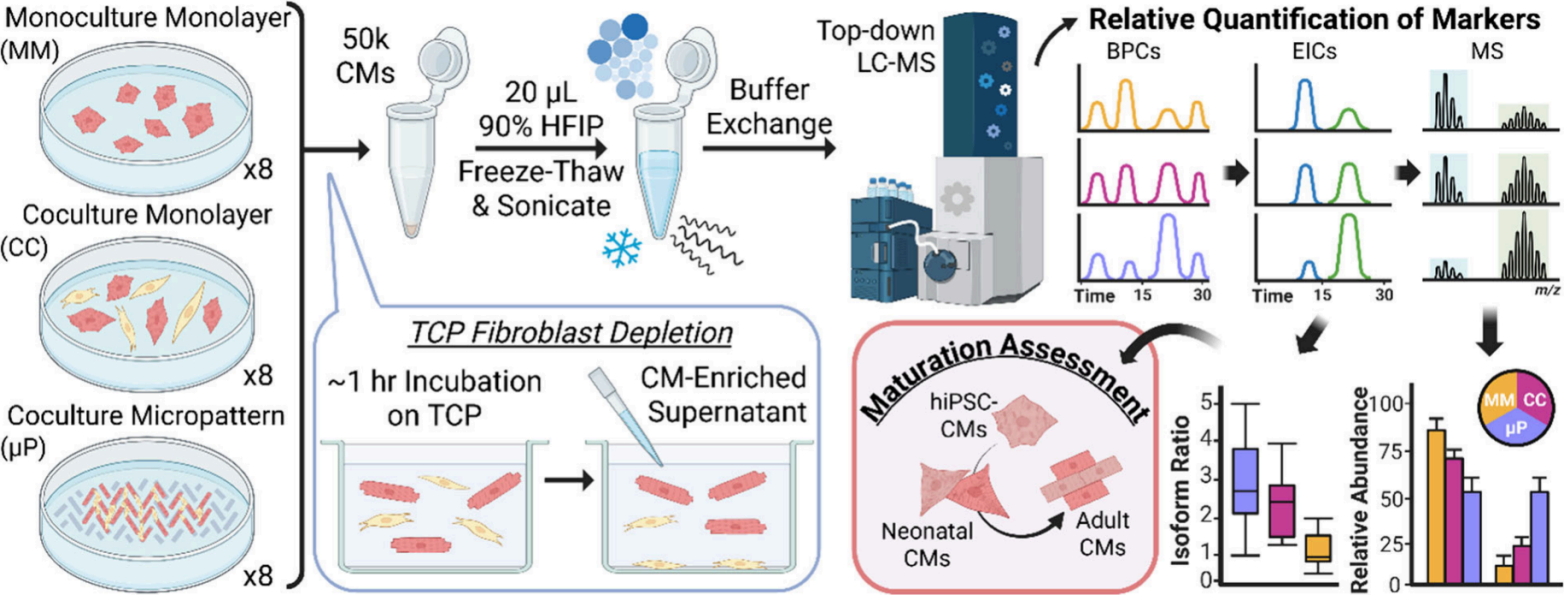
**Experimental overview for the maturation assessment of micropatterned hiPSC-CMs in comparison to hiPSC-CMs cultured on nonpatterned substrate**. Three groups (*n* = 8 each) of human induced pluripotent stem cell-derived cardiomyocytes (hiPSC-CMs) were cultured for analysis: monoculture CMs on nonpatterned substrate (MM), CMs cocultured alongside hiPSC-derived cardiac fibribroblasts (CFs) on nonpatterned substrate (CC), and CMs cocultured with CFs on the micropatterned substrate (μP). After depletion of CFs using their unique ability to quickly adhere to uncoated tissue culture plastic (TCP) plates, sarcomeric proteins were extracted from just 50,000 enriched CMs using an optimized surfactant-free protocol, followed immediately by top-down proteomic analysis.

**Figure 2:**
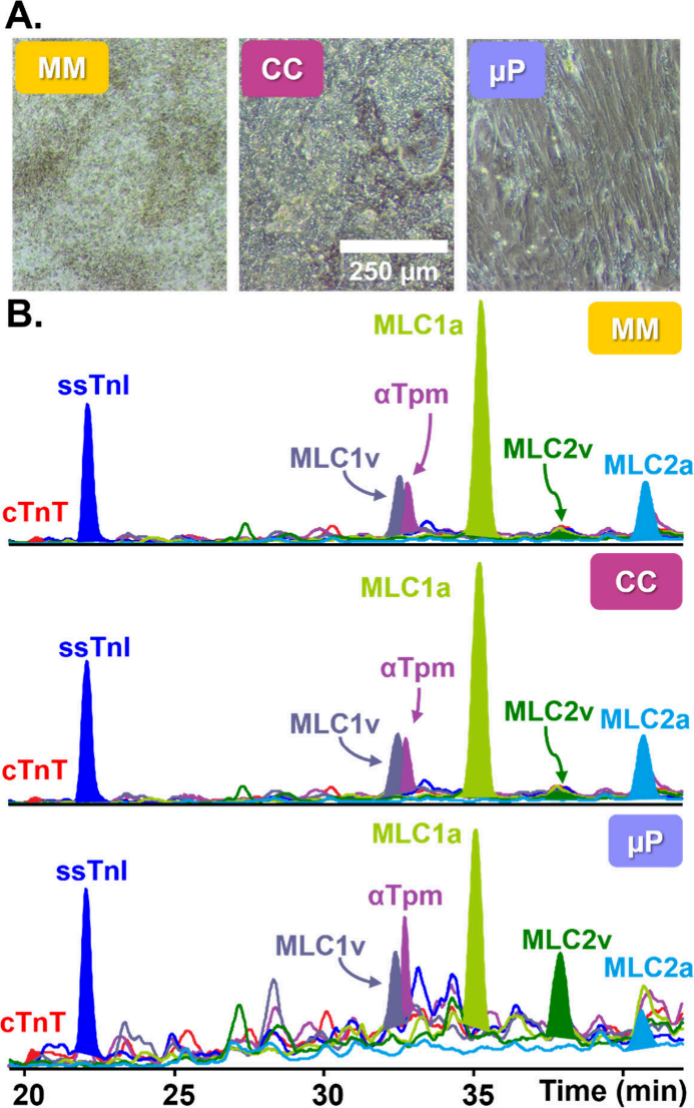
**Assessment of changes in isoform expression associated with maturation**. (A) Light microscope (10X) images of Day 14 MM, CC, and μP cultures showing the organized growth of μP hiPSC-CMs enabling elongated, rod-like cellular shapes more consistent with adult cardiomyocyte phenotypes. (B) Representative extraction ion chromatograms from MM, CC, and μP cohorts showing consistent sarcomere protein elution times along with changes in relative expression between atrial and ventricular isoforms of myosin light chain.

From this complex mixture, we successfully identified and quantified key sarcomeric proteins in each sample at the MS1 level: the canonical adult isoform of troponin T (cTnT6, *TNNT2*), slow-skeletal troponin I (ssTnI, *TNNI1*), the ventricular isoform of MLC 1 (MLC1v, *MYL3*), alpha-tropomyosin (α-Tpm, *TPM1*), the atrial isoform of MLC 1 (MLC1a, *MYL4*), the ventricular isoform of MLC 2 (MLC2v, *MYL2*), the atrial isoform of MLC 2 (MLC2a, *MYL7*), and troponin C (TnC, *TNNC1*) (Supplementary Figure S4, Table S2). The abundance of each protein is represented by the area under the curve (AUC) of their extracted ion chromatogram (EIC), and this area was used to compare the relative abundance of protein isoforms as ratios for cross-sample and cross-sample type comparison. Representative EICs of each group identified consistent intergroup elution times of sarcomere proteins while also highlighting notable changes in relative MLC isoform abundance between groups (Figure 2B). Additionally, the base peak chromatograms (BPCs), or a trace of the most abundant ion eluting from the column as a function of time, of the MM, CC, and μP groups (Supplementary Figures S5–S7) reveal consistent intragroup elution times, confirming reproducible extraction efficiency across samples and enabling reliable relative quantification within this study.

### Relative Quantification of Changes in Isoform Expression Associated with Maturation

Our previous top-down proteomics studies have demonstrated that cardiomyocyte maturation is marked by specific sarcomeric protein isoform switches.^10,29^ Specifically, we have previously found that dephosphorylation of α-Tpm and isoform switching from fetal to adult troponin isoforms such as the transition from ssTnI to cTnI, as well as cTnT’s fetal isoform (cTnT1) to the adult cTnT6. Additionally, there is a shift from atrial to ventricular MLC isoforms, specifically the transition of MLC1a and MLC2a to MLC1v and MLC2v, respectively.^10,26^ However, our data revealed that the MLCv/MLC1a ratio did not exhibit a significant change between the MM and CC samples. By contrast, a significant increase was observed in the μPs when compared to both MMs and CCs (Supplementary Figure S8). Similarly, the ratio of MLC2v/MLC2a was significantly elevated in μPs, whereas no statistically significant differences were observed when compared to MMs and CCs (Figure 3). We note that higher variability was detected in the μPs compared to MMs and CCs, likely due to the increase in model complexity inherently introducing more parameters for intersample variation.

**Figure 3:**
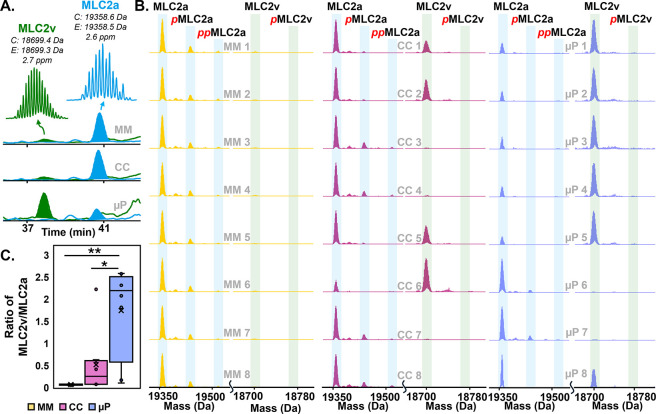
**Top-down LC-MS quantitative analysis of myosin light chain 2 ventricular and atrial isoforms**. A) Representative EICs of MM, CC, and μP cohorts displaying the relative abundance of MLC2a and MLC2v. The shaded portion indicates the AUC used for quantitation of the isoform ratio. Above each EIC, a color-matched representation of the isotopic resolution of each isoform with the calculated and experimental most abundant masses. B) Deconvoluted spectra of MLC2a and MLC2v in each sample across cohorts. C) Box plot displaying the ratio of MLC2v to MLC2a, where “n.s.” indicates a *p* > 0.05, * indicates *p* < 0.05, and ** indicates a *p* < 0.001.

In addition to the MLC isoform switching, we observed changes in the expression of cTnT isoforms across cohorts. The fetal isoform cTnT1 was absent from μPs while it was robustly expressed in MMs and CCs (Figure 4). The adult isoform of troponin T (cTnT6) was consistently expressed across all sample groups in its monophosphorylated form. Interestingly, we observed that the ratio of adult isoforms changed between culture treatments. In μPs, we identified a significant increase in the relative expression of cTnT6 to the noncanonical troponin T isoform 11 (cTnT11) compared to both MMs and CCs. In contrast, we did not observe significant differences in the cTnT6/cTnT11 ratio between the MM and CC groups. Though the ratio of these isoforms has not previously been explored as a marker of maturation in hiPSC-CMs, the absence of cTnT11 in nonfailing adult left ventricular tissue and the notably larger ratio of cTnT6/cTnT11 peaks in mass spectra (nonquantified) taken from late-stage (76 days) ECTs^10^ might suggest an unexplored association with maturation.

**Figure 4:**
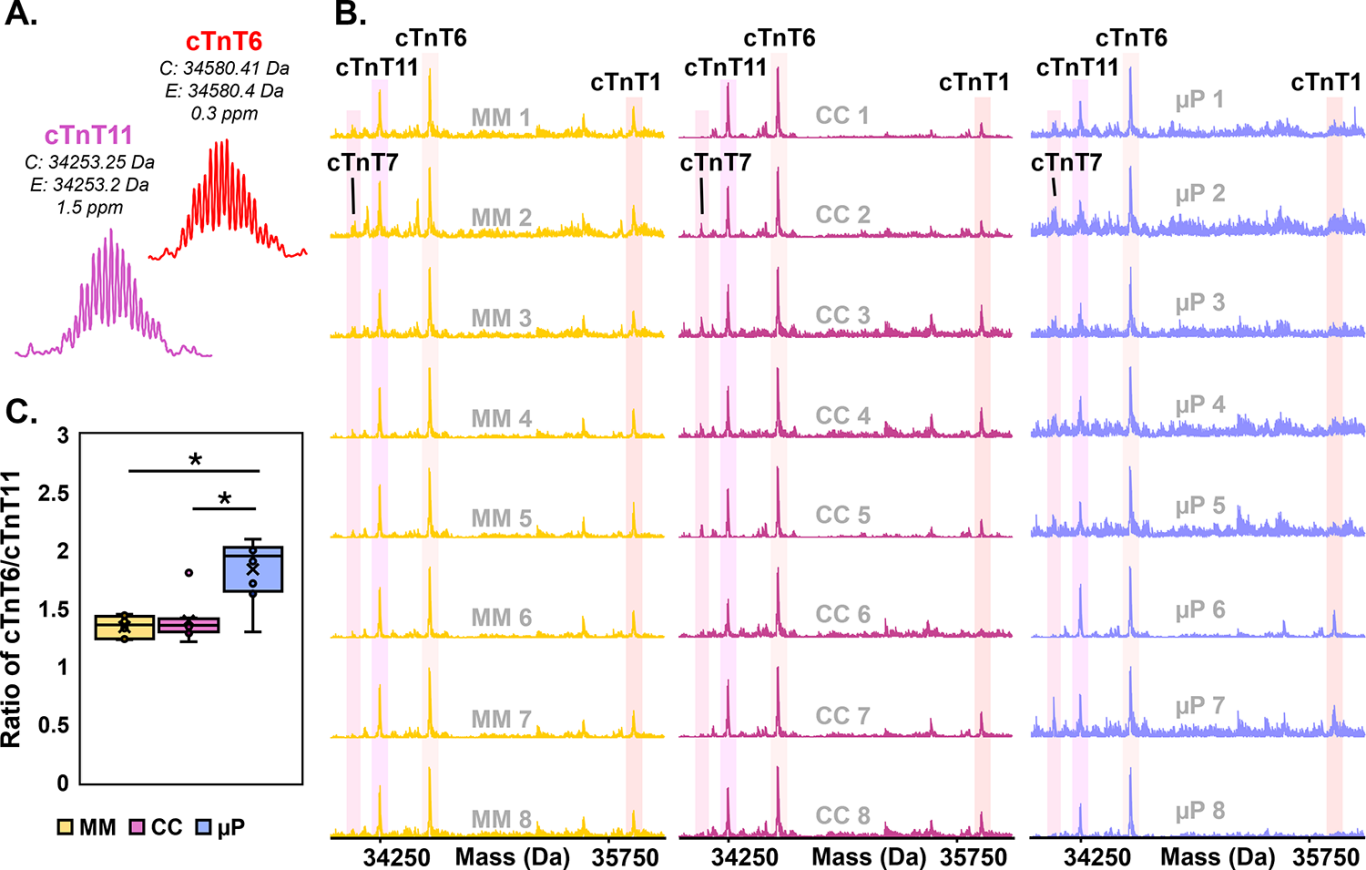
**LC-MS characterization of troponin T isoforms**. A) Representations of the isotopic resolution of troponin T canonical (cTnT6) and noncanonical (cTnT11) isoforms with the calculated and experimental most abundant masses. B) Deconvoluted spectra of cTnT isoforms in each sample across cohorts. Because cTnT isoforms coelute and have unreliable AUCs due to their low abundance, peak intensities were used for the comparison ratio. C) Box plot displaying the ratio of cTnT6 to cTnT11, where “n.s.” indicates a *p* > 0.05, * indicates *p* < 0.05, and ** indicates a *p* < 0.001.

Lastly, adult isoform cTnI was not quantifiable in any samples, though its monophosphorylated proteoform was sporadically detected at low abundances (peak signal intensity <6000) exclusively in μPs (Supplementary Figure S9). Previously, cTnI has been detected in both monoculture and coculture monolayers as well as micropatterned hiPSC-CMs using sensitive techniques such as immunoblotting and bottom-up proteomics studies.^19,29,37^ However, intact protein studies are inherently less sensitive, making low abundance proteins much harder to detect.^27^ In this study, we did not detect cTnI in MMs or CCs at any point during the data collection process. The lack of reproducible cTnI identification in μPs may reflect the biological variability in their molecular maturity, resulting in insufficiently reproducible cTnI expression for top-down analysis. Alternatively, this could suggest that the limit of detection of the instrument is insufficient to consistently detect such a low abundance proteoform in complex μP lysates. Although the absence of quantifiable cTnI implies a less mature phenotype, it is important to note that the hiPSC-CMs in this study were cultured for only 30 days, and prior studies have shown that cTnI expression primarily increases after prolonged postdifferentiation culture durations (60 days).^10,28^

### Quantitative Analysis of the Relative Abundance of Sarcomeric Protein PTMs by Top-Down Proteomics

In addition to changes in isoform abundance, this optimized protocol enabled PTM-level quantification, which we utilized to determine the relative abundance of phosphorylation in α-Tpm, MLC2a, MLC2v, and ssTnI (Figure 5). In particular, we had a specific focus on the phosphorylation of α-Tpm, an identified maturation marker.^10^ No statistically significant difference in α-Tpm phosphorylation was observed between MMs and CCs; however, μPs exhibited a significant reduction in phosphorylation relative to MMs and CCs (Supplementary Figure S10). There were also notable changes in the relative abundance of phosphorylation in the MLC2 isoforms. For MLC2a, both mono- and bisphosphorylated proteoforms (*p*MLC2a and *pp*MLC2a, respectively) were detected. A significant difference in the relative abundance of MLC2a and *pp*MLC2a was found between μPs and MMs, whereas μPs and CCs were differentially expressed only for the *p*MLC2a. The total phosphorylation of each group reveals a significant trend with decreasing total phosphorylation in the order MMs > CCs > μPs, which is consistent with previous findings that MLC2a phosphorylation decreases with increased culture time and advanced maturation.^10^ In contrast, μPs and CCs demonstrated a significant increase in MLC2v phosphorylation when compared to both MMs, with the *p*MLC2v proteoform notably absent from the MM cohort altogether. Because the phosphorylation sites of myosin light chains are populated in a cascading mechanism, a lower total phosphorylation of a MLC isoform correlates with an overall lower expression of the isoform and vice versa. Finally, the phosphorylation of ssTnI was not differentially expressed in MMs versus CCs; however, μPs had a significant decrease in the relative phosphorylation of ssTnI compared to that in both groups. The presence of monophosphorylated ssTnI and its increased abundance in more mature hiPSC-CM models was also noted in hiPSC-CMs cultured for elongated times (60 days).^10^ The biological role and prevalence of ssTnI phosphorylation has not yet been explored; however, the phosphorylation of cTnI has been shown to decrease Ca^2+^ sensitivity.^38^

**Figure 5:**
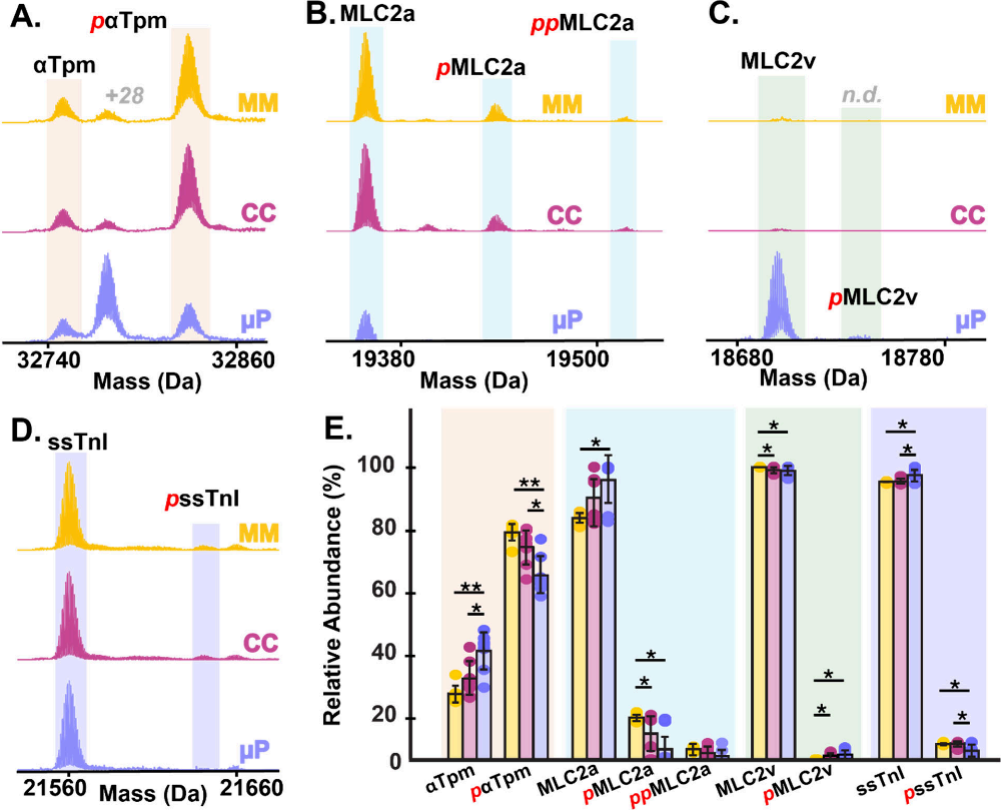
**Quantitative analysis of the relative abundance of sarcomeric protein post-translational modifications by top-down proteomics**. **(A-D)** Top-down mass spectra of sarcomere proteins alpha-Tropomyosin (α-Tpm), myosin light chain 2 atrial isoform (MLC2a), myosin light chain 2 ventricular isoform (MLC2v), and slow-skeletal troponin I (ssTnI). “n.d.” indicates that the proteoform was not detected. (E) Quantitation of proteoform relative abundance, where * indicates *p* < 0.05, and ** indicates a *p* < 0.001. The colored boxes correspond with the associated proteins highlighted in A-D.

Collectively, these findings indicate that hiPSC-CMs cocultured on micropatterns exhibit more mature molecular phenotypes than conventional monoculture and coculture monolayer hiPSC-CMs and are comparable in maturity to 3D cardiac constructs previously used in a prior proteomics study.^28^ Although this study did not include a direct side-to-side comparison of μPs with 3D constructs, our prior research has reported that 3D constructs exhibit enhanced maturity when compared with monocultured hiPSC-CMs from the same cell background through sarcomeric maturation markers.^28^ Notably, the relative abundance of phosphorylated α-Tpm in μPs was comparable to phosphorylation observed in both ESC-derived CMs cultured for 60 days^10^ and in 3D constructs (Day 53) created with the DF19–9–11T line.^28^ Further, we note that μPs exhibited a *higher* ratio of MLC1v/MLC1a than cocultured ECTs derived from the same DF19–911T line.^27^ Previous studies have shown that coculture alongside CFs accelerates sarcomere maturation of hiPSC-CM models, including micropatterns.^16,19,28^ When cocultured micropatterns were compared to CM-only micropatterns, cocultures had calcium transient (CaT) rise-up times of 30–37 ms, whereas CM-only micropatterns had CaTs of approximately 86 ms, much further from the 25 ms CaT observed in adult human left ventricular tissue.^17^ Despite this, our work indicates that neither growth on nonpatterned PDMS (in MMs) nor the addition of CFs (in CCs) induce advanced maturation during the 14 days of coculture on micropatterns (postdifferentiation), as no significant differences were observed between MMs and CCs. These findings support the conclusion that the enhanced maturation observed in μPs is primarily driven by spatial organization directed by the patterned PDMS substrate.

## Conclusions

In this study, we have established a surfactant-free protein extraction method tailored for high-sensitivity top-down proteomics of limited, heterogeneous multicell samples. We applied this method to assess the maturation of micropatterned hiPSC-CMs and observed proteoform-level maturation signatures such as the increased expression of ventricular MLC isoforms, the decreased phosphorylation of α-Tpm, and isoform switching from fetal cTnT to adult cTnT. Interestingly, we also identified an increase in the ratio of cTnT6/cTnT11 in micropatterned hiPSC-CMs, which has not yet been characterized as a marker of sarcomere maturation, potentially implicating noncanonical cTnT isoforms in a developmental role. These proteomic findings confirm that the micropatterned hiPSC-CMs exhibit more mature proteoform and isoform expression than nonpatterned cocultured and monocultured hiPSC-CMs and provide a promising platform for use in cardiac disease and arrhythmogenic studies.

## Supplementary Material







## Data Availability

Source data for this manuscript is available via MassIVE repository at massive.ucsd.edu with identifier: MSV000097864. Any additional information is available from the corresponding authors upon reasonable request.

## Notes

Author names and affiliations, table of contents (PDF)
Supplementary Methods Supplementary Figures Supplementary Figure S1. Visual representations of the experimental timeline and micropattern surface Supplementary Figure S2. Method optimization to establish surfactant-free extraction of proteins from limited number of cells Supplementary Figure S3. Technical replicates and linear instrument response analysis of the mass spectrometer Supplementary Figure S4. Top-down proteomics analysis of sarcomere proteins extracted from a representative μP sample Supplementary Figure S5. Reproducibility of monoculture monolayer biological replicates Supplementary Figure S6. Reproducibility of coculture monolayer biological replicates Supplementary Figure S7. Reproducibility of coculture micropattern biological replicates Supplementary Figure S8. Top-down LC-MS quantitation of myosin light chain 1 ventricular and atrial isoforms Supplementary Figure S9. Identification of low abundance adult cardiac troponin I (cTnI) by top-down LC-MS Supplementary Figure S10. Top-down LC-MS quantitation of alpha-Tropomyosin phosphorylation­(PDF)
Supplementary Table S1. Summary of detected proteoforms. Supplementary Table S2. Summary of identified sarcomere proteins (XLSX)
