# Supplementary material for: High-Sensitivity Top-Down Proteomics Reveals Enhanced Maturation of Micropatterned Induced Pluripotent Stem Cell-Derived Cardiomyocytes
Source: J Proteome Res. 2025 Aug 26;24(9):4335–43. doi: 10.1021/acs.jproteome.5c00505 (PMC12503376; doi:10.1021/acs.jproteome.5c00505)
Supplement: Supplementary file 1 [file pr5c00505_si_001.pdf]

SUPPORTING INFORMATION COVER PAGE

High-Sensitivity Top-Down Proteomics Reveals Enhanced Maturation of Micropatterned Induced Pluripotent Stem Cell-Derived Cardiomyocytes

Mallory C. Wilson[1,2], Mitchell Josvai[3,4], Janay K. Walters[4], Jodi Lawson[3,4], Kalina J. Rossler[2,5], Zhan Gao[2], Yanlong Zhu[2,6], Timothy J. Kamp[4], Wendy C. Crone[3,7,8], Lee L. Eckhardt[4*], and Ying Ge[1,2,6]*

[1] Department of Chemistry, University of Wisconsin-Madison, Madison, WI 53706, USA
[2] Department of Cell and Regenerative Biology, University of Wisconsin-Madison, Madison, WI 53705, USA
[3] Department of Biomedical Engineering, University of Wisconsin-Madison, Madison, WI 53706, USA
[4]Department of Medicine, School of Medicine and Public Health, University of Wisconsin-Madison, Madison, WI 53705, USA
[5]Molecular and Cellular Training Program, School of Medicine and Public Health, University of Wisconsin-Madison, Madison, WI 53705, USA
[6]Human Proteomics Program, School of Medicine and Public Health, University of Wisconsin-Madison, Madison, WI 53705, USA
[7]Department of Nuclear Engineering and Engineering Physics, University of Wisconsin-Madison, Madison, WI 53706, USA
[8]Department of Mechanical Engineering, University of Wisconsin-Madison, Madison, WI 53706, USA
*Correspondence: Dr. Ying Ge: ying.ge@wisc.edu, Dr. Lee Eckhardt: lle@medicine.wisc.edu

Supplementary Methods

Supplementary Tables and Figures
Supplementary Table S1. Summary of detected proteoforms.
Supplementary Table S2. Summary of identified sarcomere proteins.
Supplementary Figure S1. Visual representations of the experimental timeline and micropattern surface
Supplementary Figure S2. Method optimization to establish surfactant-free extraction of proteins from limited number of cells
Supplementary Figure S3. Technical replicates and linear instrument response analysis of the mass spectrometer
Supplementary Figure S4. Top-down proteomics analysis of sarcomere proteins extracted from a representative µP sample
Supplementary Figure S5. Reproducibility of monoculture monolayer biological replicates
Supplementary Figure S6. Reproducibility of coculture monolayer biological replicates
Supplementary Figure S7. Reproducibility of coculture micropattern biological replicates
Supplementary Figure S8. Top-down LC-MS quantitation of myosin light chain 1 ventricular and atrial isoforms
Supplementary Figure S9. Identification of low abundance adult cardiac troponin I (cTnI) by top-down LC-MS
Supplementary Figure S10. Top-down LC-MS quantitation of alpha-Tropomyosin phosphorylation
